# An Unusual Shockwave Complication: A Case of Type III Coronary Artery Perforation Following Intravascular Lithotripsy in a Severely Calcified Coronary Lesion

**DOI:** 10.7759/cureus.111296

**Published:** 2026-06-22

**Authors:** Amine El Houari, Wassim Beladel, Ahmad Alfay, Abdul Karim Najib, Mohamed El Minaoui

**Affiliations:** 1 Department of Cardiology, University Hospital Centre Souss Massa, Faculty of Medicine and Pharmacy, Ibn Zohr University, Agadir, MAR; 2 Department of Cardiology, Montluçon Hospital, Montluçon, FRA

**Keywords:** calcified coronary lesions, covered stent, ellis type iii coronary perforation, percutaneous coronary intervention, shockwave intravascular lithotripsy

## Abstract

Shockwave intravascular lithotripsy (S-IVL) has been increasingly adopted for the treatment of severely calcified coronary lesions, offering an innovative and controlled method of calcium modification. While the technique is generally safe, coronary artery perforation (CAP) remains a rare but life-threatening complication.

We report the case of a 71-year-old man with an extensive, long, calcified stenosis of the proximal-mid left anterior descending (LAD) artery, who underwent elective percutaneous coronary intervention (PCI) with S-IVL followed by high-pressure inflation using a non-compliant balloon. Immediately after post-dilatation, he developed an Ellis Type III LAD perforation with pericardial effusion and hemodynamic collapse. Urgent pericardiocentesis and implantation of a covered stent achieved complete sealing and restored coronary flow. The patient stabilized rapidly, was discharged without sequelae, and a follow-up optical coherence tomography (OCT) confirmed optimal stent apposition and vessel integrity. This case adds to the limited published reports of S-IVL-associated perforation, highlighting the interplay between acoustic plaque fracture and subsequent mechanical dilation in complex calcified anatomy, and emphasizing the importance of intravascular imaging and readiness for immediate complication management.

## Introduction

Coronary artery calcification represents a frequent and challenging barrier in percutaneous coronary intervention (PCI), contributing to suboptimal stent expansion, higher restenosis rates, and adverse long-term outcomes [1]. Various calcium-modifying strategies, such as high-pressure balloons, atherectomy, and scoring balloons, have been employed with variable efficacy and risk. Shockwave intravascular lithotripsy (S-IVL) has recently emerged as an innovative technology that delivers pulsatile acoustic pressure waves to selectively fracture calcified plaque, while minimizing trauma to surrounding soft tissues [2]. Prospective trials have demonstrated high procedural success and a favorable safety profile [3].

However, despite this reassuring evidence, serious complications, such as coronary artery perforation (CAP), remain possible. While large-scale clinical trials (e.g., the DISRUPT CAD series) reported no Ellis Type III perforations directly attributable to IVL, post-marketing data and isolated case reports suggest that such events, though rare, can occur in real-world practice [4,5].

Ellis’s classification remains the most widely accepted system to categorize CAP severity, with Type III perforations representing the most critical form [6,7]. These are characterized by frank perforation with active contrast extravasation into the pericardial space or adjacent cardiac chambers, and are most associated with adverse outcomes, such as cardiac tamponade and the need for emergency surgical intervention [6,7]. 

In this report, we present a case of Ellis Type III LAD (left anterior descending) perforation following S-IVL in a heavily calcified coronary lesion, underscoring the importance of careful procedural planning, recognition of complications, and optimal management strategies.

## Case presentation

A 71-year-old man was transferred to the Intensive Cardiac Care Unit following stabilization in the Intensive Care Unit for an acute exacerbation of severe chronic obstructive pulmonary disease. His past medical history included well-controlled Type 2 diabetes mellitus, hypertension, dyslipidemia, chronic obstructive pulmonary disease, and significant peripheral arterial disease, with prior bilateral carotid endarterectomy and right iliac artery stenting for occlusive lower extremity disease. His family history was negative for premature cardiovascular disease, and he reported no history of smoking, alcohol use, or illicit drug use. The patient’s long-term medications included metformin 850 mg once daily, gliclazide 60 mg once daily, perindopril/indapamide 10/2.5 mg once daily, rilmenidine 1 mg once daily, rosuvastatin 5 mg once daily, aspirin 100 mg once daily, and inhaled fluticasone furoate/vilanterol once daily.

On admission, the patient was conscious, normotensive, and eupneic, with no clinical evidence of cardiac decompensation. His electrocardiogram showed sinus rhythm with signs of left ventricular hypertrophy, left bundle branch block morphology, and frequent premature ventricular complexes. Transthoracic echocardiography demonstrated a moderately reduced left ventricular ejection fraction, estimated at 45%, with regional wall motion abnormalities and no significant valvular pathology. The patient had a history of stable angina of several weeks’ duration, typically exertional, triggered by moderate physical activity, and relieved by rest; symptoms had remained stable without recent progression. A recently performed cardiac computed tomography (CT) calcium scoring study revealed a markedly elevated Agatston score of 4000. Given this context, diagnostic coronary angiography was indicated for further evaluation of coronary anatomy.

**Figure 1:**
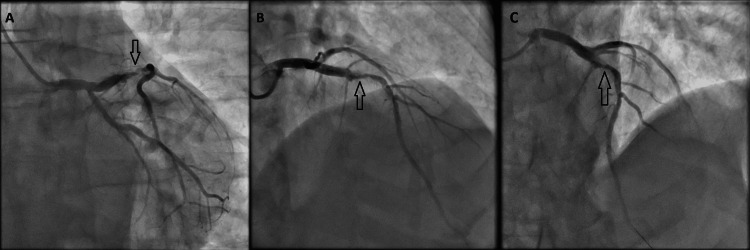
Multiview Coronary Angiography of the LAD Artery (A) RAO cranial view showing a complex, heavily calcified stenosis in the proximal to mid LAD (arrow). (B) PA cranial view confirming the lesion's severity and extent (arrow). (C) LAO cranial view providing additional anatomical detail of the calcified segment (arrow). RAO, Right Anterior Oblique; PA, Posteroanterior; LAO, Left Anterior Oblique; LAD, Left Anterior Descending

Coronary angiography revealed a complex, long (>20 mm), and heavily calcified, concentric lesion in the proximal to mid LAD artery, classified as a Type C stenosis with 70%-90% luminal narrowing and involving the takeoff of the first diagonal branch (Figure 1). The reference vessel diameter was approximately 4.5 mm, and coronary flow was impaired (Thrombolysis in Myocardial Infarction (TIMI) 2). The left main coronary artery was free of significant disease. The left circumflex artery showed only mild irregularities without significant stenosis. The right coronary artery (RCA) was mildly calcified, with a long, intermediate Type C stenosis of about 50% in its proximal to mid segment, with a reference diameter of 3.5 mm and preserved TIMI 3 flow. The syntax score was evaluated at 21. No pre-procedural optical coherence tomography (OCT) or intravascular ultrasound (IVUS) was performed.

Following the Heart Team discussion, the decision was made to proceed with PCI of the LAD. The procedure was performed under standard anticoagulation, with a single intravenous bolus of unfractionated heparin 5000 IU at the start of the intervention. A loading dose of clopidogrel (300 mg orally) was administered in the catheterization laboratory before angioplasty. No additional antithrombotic agents were used. Pre-procedural coagulation tests, including prothrombin time and activated partial thromboplastin time, were within normal limits.

The procedure was performed via right radial artery access using a 6F JL3.5 Medtronic Launcher guiding catheter (Medtronic, Inc., Minneapolis, MN, USA). A 3.0 × 15 mm non-compliant balloon was first used to pre-dilate the lesion and confirm its calcified and resistant nature. A 3.5 × 12 mm Shockwave C2+ IVL balloon (Shockwave Medical, Inc., Santa Clara, CA, USA) was then advanced over a SION 0.014” guidewire (ASAHI INTECC Co., Ltd., Aichi, Japan) to the lesion site. Lithotripsy was performed in eight cycles, each comprising 10 acoustic pulses. For each cycle, the IVL balloon was inflated to 4 atm for apposition, followed by inflation to 6 atm for a few seconds before deflation, totaling 80 pulses. To optimize luminal gain, post-dilatation was attempted with a 4.5 × 12 mm non-compliant balloon at 12 atm for 10 seconds.

**Figure 2:**
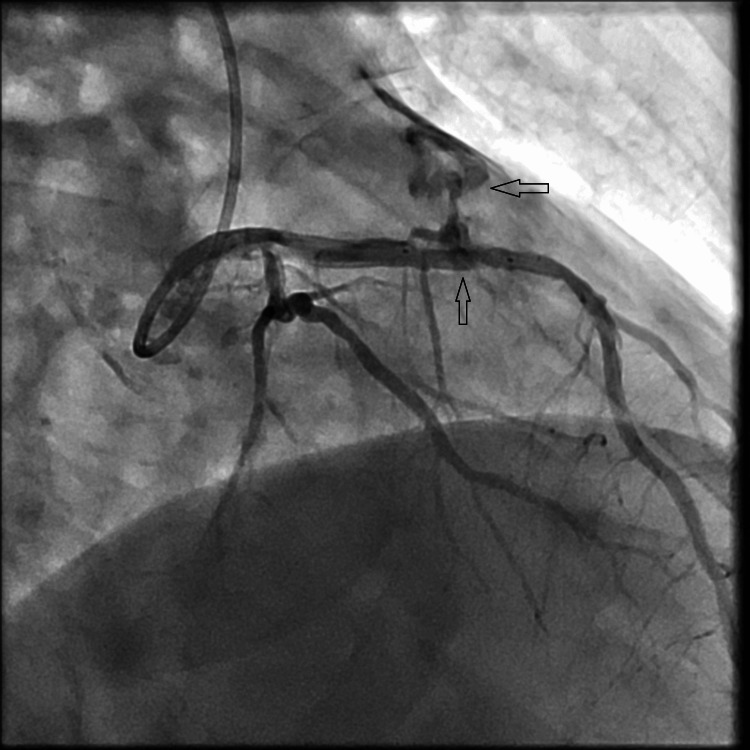
Coronary Angiography Revealing Type III Ellis Coronary Perforation Angiographic frame showing active contrast extravasation into the pericardial space (arrows), consistent with a Type III Ellis coronary perforation of the proximal LAD following high-pressure post-dilatation. LAD, Left Anterior Descending

Immediately after the final balloon inflation, the patient developed severe chest pain and hypotension. Emergency coronary injection revealed active contrast extravasation into the pericardial space, consistent with a Type III Ellis coronary perforation (Figure 2).

**Figure 3:**
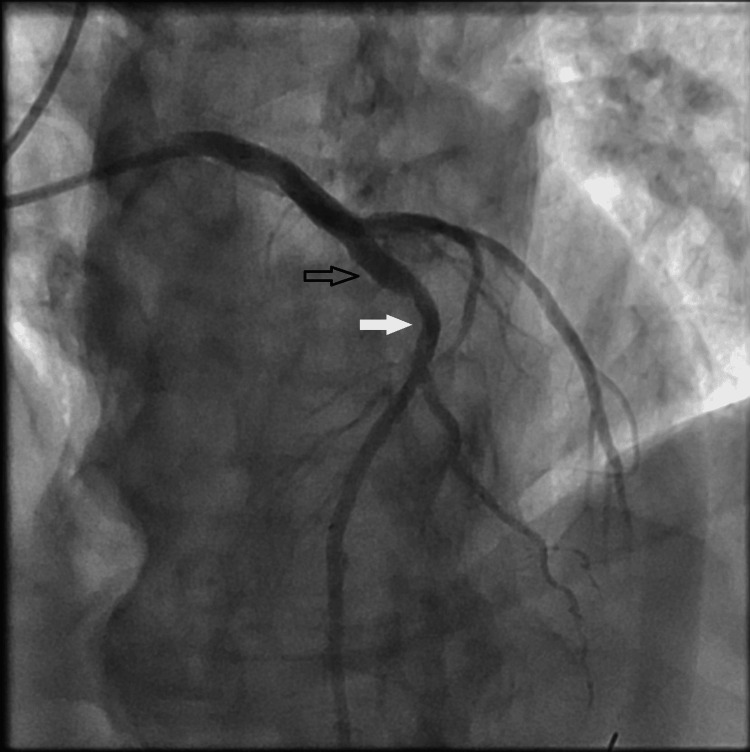
Sealing of Coronary Perforation With Covered Stent Angiographic image showing complete sealing of the proximal LAD perforation with a covered stent (black arrow) and a residual stenotic lesion distal to the implanted stent (white arrow). LAD, Left Anterior Descending

Bedside echocardiography confirmed a moderate pericardial effusion. Given the hemodynamic compromise, emergent pericardiocentesis was performed first, draining 300 mL of hemorrhagic fluid and stabilizing the patient. Subsequently, the site of perforation was successfully sealed with a 4.5 × 20 mm Papyrus-covered stent (BIOTRONIK SE & Co. KG, Berlin, Germany), restoring full coronary flow and hemodynamic stability (Figure 3).

**Figure 4:**
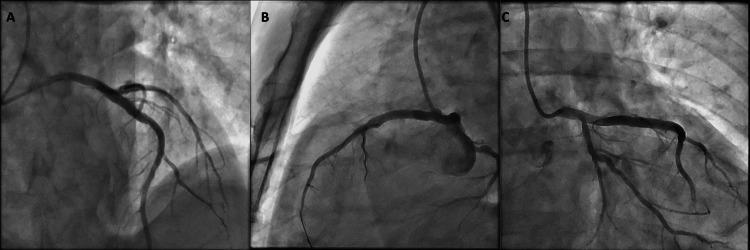
Final Angiographic Result of LAD PCI Coronary angiography views illustrating the final outcome in the previously ruptured segment of the proximal LAD, after covered stent implantation and completion with an additional drug-eluting stent placed distally. (A) RAO cranial view showing complete sealing of the rupture site and full stent expansion. (B) LAO caudal view confirming optimal vessel restoration and apposition of both stents. (C) RAO caudal view demonstrating uninterrupted distal flow and no residual stenosis. RAO, Right Anterior Oblique; LAO, Left Anterior Oblique; LAD, Left Anterior Descending; PCI, Percutaneous Coronary Intervention

To complete the treatment of the LAD lesion, a 3.5 × 28 mm Synergy Megatron drug-eluting stent (Boston Scientific, Marlborough, MA, USA) was implanted immediately distal to the covered stent, ensuring full lesion coverage and achieving an optimal final angiographic result (Figure 4).

The patient was transferred to the Intensive Care Unit for monitoring. He remained hemodynamically stable, with no recurrence of effusion. Dual antiplatelet therapy (aspirin plus clopidogrel, with gastric protection) was initiated in addition to his baseline medications. He was discharged five days later in good clinical condition.

**Figure 5:**
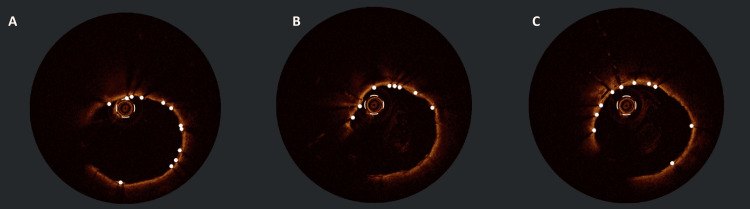
OCT Images of the Previously Perforated LAD Segment Treated With a Covered Stent OCT images obtained at different levels of the previously perforated proximal LAD segment after covered stent implantation. (A) Proximal portion of the stented segment showing optimal stent apposition and intact vessel architecture. (B) Mid-stent view confirming full coverage of the perforated site with no evidence of malapposition or intramural hematoma. (C) Distal stent edge demonstrating satisfactory integration into the vessel wall and absence of residual structural abnormalities. OCT, Optical Coherence Tomography; LAD, Left Anterior Descending

During a subsequent procedure performed 40 days later for RCA revascularization, OCT was performed at the previously treated LAD site, confirming excellent stent apposition and the absence of residual perforation or late complications (Figure 5).

At three-month follow-up, the patient remained asymptomatic, with preserved left ventricular systolic function and no recurrence of pericardial effusion, demonstrating a favorable short-term outcome following this high-risk intervention.

## Discussion

The incidence of CAP during PCI ranges between 0.1% and 3%, but rises significantly in complex lesions, including chronic total occlusions (CTOs), heavily calcified plaques, and tortuous segments [7,8]. Ellis Type III CAP is rare and represents the most clinically severe form of CAP, with an estimated incidence of approximately 0.23% [9]. It is frequently associated with rapid hemodynamic compromise and carries significant morbidity and mortality, with reported tamponade rates ranging from 10% to 60%, surgical intervention required in 20% to 40% of cases, and mortality reaching as high as 44% [9].

S-IVL, while designed to minimize barotrauma by utilizing low-pressure balloon inflation, is not exempt from causing CAP, particularly in anatomically vulnerable segments. According to reports from the MAUDE (Manufacturer and User Facility Device Experience) database and single-center experiences, the incidence of CAP associated with S-IVL ranges from 0.2% to 1.8%, with perforations predominantly occurring in the setting of complex lesions or off-label use [5,10,11].

CAP during S-IVL is probably multifactorial, often resulting from a combination of procedural, anatomical, and mechanical factors. The acoustic pressure waves emitted by the IVL balloon are designed to selectively fracture calcified plaques; however, these calcium fractures can sometimes compromise the structural integrity of the vessel wall, rendering it more susceptible to rupture [4,12-14]. Moreover, the adjunctive use of other interventional techniques - such as advanced guidewires, high-pressure or cutting balloons, improved stent platforms, and rotational atherectomy - has been associated with an increased risk of vessel injury and perforation [4,12-14]. In addition, the anatomical complexity of the target lesion plays a significant role. Specifically, lesions with severe tortuosity, asymmetrical or nodular calcium deposits, and CTOs present higher mechanical stress during intervention and are well-recognized risk factors for CAP [4,12-14].

In our patient, the sequence of high-pressure balloon inflation following S-IVL may have acted synergistically to predispose to vessel rupture. It is plausible that acoustic energy delivered during lithotripsy may have weakened the arterial wall by fracturing the calcium arc, and subsequent mechanical expansion at high pressure with a non-compliant balloon exacerbated the vulnerability, leading to frank perforation. This temporal and mechanistic association emphasizes the need for caution when combining techniques, and supports individualized planning guided by intravascular imaging.

To mitigate these risks, the use of intracoronary imaging modalities such as IVUS or OCT has been recommended [4,15]. These tools facilitate precise assessment of lesion morphology, guide appropriate device selection, and help optimize procedural strategies - ultimately reducing the likelihood of perforation [4,15].

Management of Ellis Type III CAP requires rapid recognition and a structured approach due to its association with hemodynamic instability and high mortality. The primary goal is to seal the site of extravasation and manage complications such as tamponade. In this case, emergent pericardiocentesis was performed concurrently with covered stent deployment to stabilize the patient. Prolonged low-pressure balloon inflation is often the initial step to achieve hemostasis while preserving distal perfusion [9]. If ineffective, covered stents - particularly polytetrafluoroethylene-covered devices - are the preferred option due to their high success rate in sealing large perforations, although their bulky profile can pose delivery challenges in tortuous or calcified vessels [9]. In cases where stent placement is not feasible or fails, embolization with microcoils or other agents may be considered, especially in distal vessel perforations [9]. Pericardiocentesis is essential in the presence of tamponade, and reversal of anticoagulation should be considered based on the severity of bleeding and the presence of a stent [9]. If conservative and percutaneous measures fail or tamponade persists, emergent surgical intervention may be necessary [9].

At three-month follow-up, the patient remained asymptomatic, with preserved left ventricular function and no recurrence of pericardial effusion, highlighting the efficacy and safety of prompt percutaneous management in this high-risk scenario.

As the use of S-IVL expands, further data from registries and real-world studies are necessary to refine patient selection criteria and procedural protocols to mitigate risks. Comparative effectiveness studies between S-IVL and other calcium modification technologies, including rotational atherectomy and orbital atherectomy, are also warranted to establish best practices [16]. Additionally, operator experience and institutional preparedness for managing CAP critically influence patient outcomes, underscoring the need for multidisciplinary teams and hybrid catheterization suites [16].

Finally, this report represents a single case, and conclusions should be interpreted with caution; however, it highlights key considerations in the prevention, recognition, and management of S-IVL-associated coronary perforation.

## Conclusions

Our case report illustrates that, despite its generally favorable safety profile, S-IVL can be associated with serious complications such as Ellis Type III coronary perforation, particularly in complex, long, heavily calcified lesions treated with adjunctive high-pressure balloon angioplasty. The interaction between acoustic plaque modification and subsequent mechanical dilation may increase the risk of vessel rupture. This case underscores the importance of meticulous procedural planning, including the use of intravascular imaging for lesion assessment, careful selection of adjunctive devices, and readiness for emergent management to ensure safe and effective outcomes in high-risk PCI.
